# Supplementary material for: Neurocognitive profile in HIV subjects on INSTI-regimen- one year follow up: Is there room for optimism?
Source: PLoS One. 2024 Jun 26;19(6):e0306278. doi: 10.1371/journal.pone.0306278 (PMC11207154; doi:10.1371/journal.pone.0306278)
Supplement: Attachment — Submitted filename: Response to Reviewers.docx [file pone.0306278.s002.docx]

Response to Reviewers

**Journal Requirements:**

When submitting your revision, we need you to address these additional requirements.

1. Please ensure that your manuscript meets PLOS ONE's style requirements, including those for file naming. The PLOS ONE style templates can be found at
   <https://journals.plos.org/plosone/s/file?id=wjVg/PLOSOne_formatting_sample_main_body.pdf> and 
   <https://journals.plos.org/plosone/s/file?id=ba62/PLOSOne_formatting_sample_title_authors_affiliations.pdf>

The complete work has now been prepared according to the instructions in the sent documents. Thanks for the suggestions.

2. Please note that funding information should not appear in any section or other areas of your manuscript. We will only publish funding information present in the Funding Statement section of the online submission form. Please remove any funding-related text from the manuscript.

Funding information has been removed from the manuscript.

3. We note that the grant information you provided in the ‘Funding Information’ and ‘Financial Disclosure’ sections do not match.

When you resubmit, please ensure that you provide the correct grant numbers for the awards you received for your study in the ‘Funding Information’ section.

Thanks for the correction; we have corrected the error.

4. Thank you for stating the following financial disclosure: 
   "This work is supported in part by a research grant from the Investigator-Initiated Studies Program of Merck Sharp & Dohme doo."

Please state what role the funders took in the study.  If the funders had no role, please state: "The funders had no role in study design, data collection and analysis, decision to publish, or preparation of the manuscript." 
If this statement is not correct you must amend it as needed. 
Please include this amended Role of Funder statement in your cover letter; we will change the online submission form on your behalf.

We have supplemented the statement in the requested manner.

5. For studies involving third-party data, we encourage authors to share any data specific to their analyses that they can legally distribute. PLOS recognizes, however, that authors may be using third-party data they do not have the rights to share. When third-party data cannot be publicly shared, authors must provide all information necessary for interested researchers to apply to gain access to the data. (<https://journals.plos.org/plosone/s/data-availability#loc-acceptable-data-access-restrictions>)

For any third-party data that the authors cannot legally distribute, they should include the following information in their Data Availability Statement upon submission:
1) A description of the data set and the third-party source
2) If applicable, verification of permission to use the data set
3) Confirmation of whether the authors received any special privileges in accessing the data that other researchers would not have
4) All necessary contact information others would need to apply to gain access to the data

I once again checked the issue of the possibility of sharing and having open data.

The results of the study of which this paper is a part exist site clinical trials. I am sending the registration statement as a document.

I asked our sponsors and received an answer: “The PI owns the data for this study as it is considered a non-Merck study with the institution as the sponsor. The collected data belong to the Faculty of Medicine research team in Novi Sad. If necessary, we can share the data with the protection of the respondents' personal data.

6. When completing the data availability statement of the submission form, you indicated that you will make your data available on acceptance. We strongly recommend all authors decide on a data sharing plan before acceptance, as the process can be lengthy and hold up publication timelines. Please note that, though access restrictions are acceptable now, your entire data will need to be made freely accessible if your manuscript is accepted for publication. This policy applies to all data except where public deposition would breach compliance with the protocol approved by your research ethics board. If you are unable to adhere to our open data policy, please kindly revise your statement to explain your reasoning and we will seek the editor's input on an exemption. Please be assured that, once you have provided your new statement, the assessment of your exemption will not hold up the peer review process.

Answered in the previous section.

7. Your ethics statement should only appear in the Methods section of your manuscript. If your ethics statement is written in any section besides the Methods, please move it to the Methods section and delete it from any other section. Please ensure that your ethics statement is included in your manuscript, as the ethics statement entered into the online submission form will not be published alongside your manuscript.

We have moved the ethics statement in the Methods section.

**Comments to the Author**

Reviewer #1: This is an interesting study. The major limitations are the short time frame for repeat testing, the small number of patients with HAND and the lack of any power analyses to address the issue of whether INSTI drug regimens can protect or treat mild HIV associated neurocognitive disorder

Thank you for the positive evaluation. We agree with the study's recognised limitations. We included and emphasised all of the above in the section dealing with the limitations of this research and recommendations for future research.

Reviewer #2: PONE-D-24-09779 compared the effects of RAL and DTG on neurocognition among newly diagnosed PWH during a one-year follow-up. The conclusion was no significant difference between these regimens. In general, the MS was well written with sound introduction, detailed statistical analyses, and discussion. My comments are brief on certain critical information that is missing in the MS.

Thank you for the positive evaluation.

1. The concurrent drug use including substances should be included. Certain medications and many substances have a direct impact on neurocognition. Thus, this information is critical.

Thanks for such a useful suggestion. We entered data on the uniformity of the group in the sense of not taking any additional therapy for diseases other than HIV. We also entered the data and checked the correlations with the backbone therapy that the subjects were taking. There were three types of backbone therapy in the study: tenofovir, abacavir and lamivudine, whose effect was controlled.

2. The sample size calculation is missing. It is unclear how this sample size in each group was determined to detect the difference in cognition between the study groups. At least a discussion of power analysis should be provided.

We included the sample size calculation and the fact that the sample size is one of this research's limitations.

3. A discussion of additional domain specific cognitive assessment to MoCA should be considered, particularly the rationales and benefits from those assessments.

Slazemo se da bi bilo zanimljivo razmatrati specificne domene MoCA testa. U ovom istraživanju smo ovaj test koristili kao trijažni test za pojavu HANDa a određene specifične domene pokrili smo dodatnim testovima u celosti (brojanje, verbal fluency...). Predlog za analizu specificnih domena MoCA testa smo naveli kao preporuku za buduca istrazivanja.

We agree that it would be interesting to consider specific domains of the MoCA test. In this research, we used this test as a triage test for the appearance of HAND, and we covered certain specific domains with additional tests in their entirety (counting, verbal fluency...). We have listed the proposal for analysing specific domains of the MoCA test as a recommendation for future research.

4. The details of RAL and DTG are missing, including dosage, treatment interval and drug combinations.

We included the length of therapy and backbone therapy as control variables in the manuscript.

We do not have data on patient doses, so we have added that to the section on the study's limitations.

Many thanks for the extremely useful suggestions for improving the work.
